# Rise and Persistence of Global M1T1 Clone of *Streptococcus pyogenes*

**DOI:** 10.3201/eid1410.071660

**Published:** 2008-10

**Authors:** Ramy K. Aziz, Malak Kotb

**Affiliations:** Cairo University, Cairo, Egypt (R.K. Aziz); VA Medical Center, Memphis, Tennessee, USA (R.K. Aziz, M. Kotb); University of Tennessee Health Science Center, Memphis (M. Kotb); University of Cincinnati, Cincinnati, Ohio, USA (M. Kotb)

**Keywords:** M1T1 strain, Streptococcus pyogenes, epidemiology, strain diversification, invasive, pathogenomics, phage mobilization, horizontal gene transfer, perspective

## Abstract

The resurgence of severe invasive group A streptococcal infections in the 1980s is a typical example of the reemergence of an infectious disease. We found that this resurgence is a consequence of the diversification of particular strains of the bacteria. Among these strains is a highly virulent subclone of serotype M1T1 that has exhibited unusual epidemiologic features and virulence, unlike all other streptococcal strains. This clonal strain, commonly isolated from both noninvasive and invasive infection cases, is most frequently associated with severe invasive diseases. Because of its unusual prevalence, global spread, and increased virulence, we investigated the unique features that likely confer its unusual properties. In doing so, we found that the increased virulence of this clonal strain can be attributed to its diversification through phage mobilization and its ability to sense and adapt to different host environments; accordingly, the fittest members of this diverse bacterial community are selected to survive and invade host tissue.

## Abstract 2

M1T1 strain, its diversification by phage acquisition, and the in vivo selection of more fit members of its community present an intriguing example of the emergence of hypervirulent forms of a human pathogen.

Group A streptococci (GAS or *Streptococcus pyogenes*) are strictly human pathogens that normally colonize the throat or skin without causing disease. Members of this species are differentiated into >100 types on the basis of immunogenic differences in their surface M proteins and polymorphisms in the *emm* gene (*1*). The range of GAS diseases is broad and includes both localized and systemic infections that can cause acute or chronic illnesses (Table 1 in Technical Appendix). In most cases, these bacteria cause pharyngitis (sore throat), tonsillitis, or skin infections such as impetigo/pyoderma. At times, however, the bacteria gain access to normally sterile sites and cause invasive disease. Depending on complex host–pathogen interactions, invasive GAS infections can cause either severe shock and multiple organ failure or nonsevere systemic disease, e.g., mild bacteremia and cellulitis (*2*,*3*). Likewise, invasive infections of soft tissues can be severe, e.g., necrotizing fasciitis (NF), or nonsevere, e.g., cellulitis or erysipelas (*4*). Whereas host genetic susceptibility plays a key role in modulating disease manifestation, variations in bacterial virulence properties contribute to infection severity.

Despite reports that particular serotypes or *emm* types are more commonly associated than others with particular disease manifestations, serotypic designation does not always reflect the pathogenic potential of a given strain. As we discuss below, serotype diversification can convert relatively avirulent serotypes to highly virulent ones. Dissection of molecular and genetic events leading to such diversification provides insight into how the changes in pathogenesis and host–pathogen interactions can lead to the resurgence of a severe infectious disease.

## Resurgence of Severe Invasive Streptococcal Diseases and Emergence of Highly Virulent GAS Strains

In the 19th century, GAS infections were associated with severe and frequent epidemics of invasive and often fatal illnesses, including a pandemic of scarlet fever in the United States and Great Britain (*5*). Invasive GAS infections with severe manifestations continued through the 1920s (*5*). The severity of these illnesses then declined notably until the early 1980s, when a statistically significant simultaneous recrudescence of the severe and fatal forms of invasive GAS infections occurred in different parts of the industrialized world (*6*,*7*). Accordingly, in 1993 a working group developed the case definition for streptococcal toxic-shock syndrome (STSS) as hypotension accompanied by multiple organ failure, indicated by 2 of the following signs: renal impairment, coagulopathy, liver involvement, adult respiratory distress syndrome, a generalized rash, and soft tissue necrosis (*8*). A modified definition of STSS was later adopted to focus on the host immune-mediated severe systemic disease associated with invasive infections, manifested by hypotension and multiple organ failure, excluding skin rash, soft-tissue necrosis, and gangrene (*2*). Similarly, NF was defined by the histopathologic identification of necrosis of superficial fascia and a polymorphonuclear infiltrate and edema of the reticular dermis, subcutaneous fat, and superficial fascia (*9*,*10*). The speed and rigor by which invasive GAS infections spread in the host, sometimes causing severe damage to the fascia and muscles, prompted its designation as the “flesh-eating disease.”

Epidemiologic studies showed that the resurgence of severe invasive GAS infection was not limited to sporadic cases; rather, it represented a global spread, ushering in a new pandemic, similar to that reported in the earlier part of the 20th century. An important feature of this latest pandemic is its association with a distinct epidemiologic shift in GAS serotypes. Although many GAS serotypes are capable of causing severe diseases, a few were more frequently isolated from patients with severe cases, e.g., M1, M3, M18, and M28 strains (Table 2 in Technical Appendix). However, whether those serotypes cause more severe disease because of their hypervirulence or because they were also the most prevalently isolated strains in the community at that time was not clear (*11*,*12*). These possibilities are not mutually exclusive, but in fact may be related. We believe that the unique features of the newly emerged subclones of GAS serotypes, in particular the M1T1 clonal strain, evolved as a result of diversification of the bacteria and acquisition of new genes that improved their fitness to infect humans. This, together with host-imposed pressure, resulted in the selection of hypervirulent mutants of this strain associated with an ability to cause severe forms of the invasive infection in susceptible persons.

## Features of the Newly Emerged Hypervirulent Global M1T1 Strain

Whereas most GAS serotypes traditionally exhibit cyclic epidemiologic patterns, appearing and disappearing from the community at different times (*13*), the M1T1 subclone has persisted globally for more than a quarter of a century as the most frequently isolated serotype from patients with invasive and noninvasive cases. Advanced molecular and genomic tools showed a great deal of diversity among GAS strains belonging to the same serotype, and the M1 serotype is no exception. The clonality of the reemerged M1T1 strain was first described by Cleary et al. (*14*) and later confirmed by others by the use of different molecular methods (Table 3 in Technical Appendix), which confirmed that the M1T1 clone differs from its ancestral M1 clone in several aspects. We will present evidence that those differences have indeed contributed to the stark difference in epidemiologic and virulence properties between 2 strains belonging to the same serotype.

Together with the Ontario Streptococcal Study Group and the Centers for Disease Control and Prevention, our laboratory launched one of the earliest and most comprehensive prospective studies of invasive GAS pathogenesis in Ontario (*8*), where active surveillance of invasive GAS cases took place during 1992–2002 (*4*,*9*). M1T1 isolates recovered from patients with noninvasive as well as invasive cases, of varying severity, were extensively analyzed at the molecular level and shown to be clonal regardless of case severity (*3*). This clonal M1T1 strain possesses the *emm1.0* allele of the M1 gene (*3*) and is one of the opacity factor–negative GAS serotypes. This strain differs in its virulence and genomic content from other less virulent M1 strains, represented by strain M1 SF370, the first fully sequenced GAS strain (*15*).

Several events appear to have contributed to the diversification of the M1 GAS serotype, leading to the emergence of the M1T1 global strain. Specifically, diversification through the loss and/or acquisition of phages that took away certain genes and introduced new ones into the M1 serotype is a major contributor to the emergence of this strain. This phenomenon is certainly not unique to the M1T1 strain, but is also seen in the M3T3 and M18 strains (*16*,*17*), which co-emerged with the M1T1 clonal strain in the 1980s.

## Contribution of Prophages to Emergence of Global M1T1 Strain

In 1996, Cleary et al. found that the globally disseminated M1T1 differs from its closely related M1 subtypes by 70 kb of phage DNA (*18*). Ensuing studies from our group, in which we conducted global genomic comparison of the M1T1 clones and the closely related M1 SF370 strain, demonstrated that most of the genetic differences (≈5% divergence) were accounted for by phage or phagelike sequences. After assembling these distinct sequences, we identified 2 novel prophages that were introduced into the M1T1 global strain (*19*). One prophage (SPhinX) carries the *speA2* gene, which encodes the potent superantigen SpeA; the other (PhiRamid) carries the *sda1* gene, which encodes the most potent streptococcal nuclease identified thus far (*19*,*20*). The introduction of these phages into the M1T1 clonal strain was later confirmed by the complete genome sequence of a clinical M1T1 isolate, MGAS5005 (*21*).

**Figure 1:**
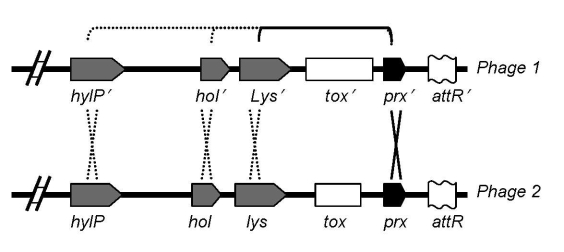
Suggested model for toxin mobilization between phages, reprinted from Aziz et al. (*19*). Recombination hot spots on both sides of the toxin genes are shown: one is *prx* (paratox), and the other may be *lys* (lysin), *hol* (holin), or *hylP* (phage hyaluronidase).

**Figure 2:**
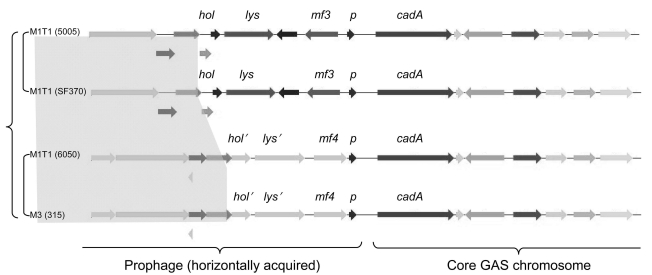
Similarities and differences between the 4 highly related prophages 5005.2, 370.3, MemPhiS, and 315.3. The figure, generated by the SEED comparison tools (*22*) (http://theseed.uchicago.edu), shows the physical maps of the 4 prophages near their attachment sites. Arrows with identical colors designate orthologous genes; those in gray designate alternative alleles of the genes. *p*, *prx; mf*, mitogenic factor; *cadA*, heavy metal/cadmium transporter ATPase; GAS, group A streptococci.

The M1T1 prophages exhibit considerable genetic mosaicism, and the sequence analysis of the 2 novel M1T1 phages demonstrates that these bacterial viruses continuously exchange functional modules by various genetic mechanisms, including different modes of recombination (*19*). We believe that exchange between the lysis and lysogenic conversion modules of GAS prophages has led to the swapping of virulence genes (toxins) among phages (*19*). We also believe that this process is facilitated by a highly conserved gene, paratox (*prx*), commonly found between the toxin gene and phage attachment site. Conserved *prx* sequences on 1 side of the toxin gene together with 1–3 highly conserved phage genes on the other side (lysin, holin, and/or hyaluronidase genes) are likely to facilitate recombination events leading to swapping of toxin genes among bacterial isolates (Figure 1) (*19*). This notion is supported by the fact that strains belonging to the same serotype may have different virulence components carried by the same or highly similar phages, whereas those belonging to different serotypes may, in fact, have identical phage-encoded toxins. For example, 4 highly similar phages (370.3, 5005.2, MemPhiS, 315.3) identified in M1 SF370, M1T1 5005, M1T1 6050, and M3 strains, respectively, have different DNases in their lysogenic conversion modules. Phages 370.3 and 5005.2 are >99% identical to each other and carry the *mf3* gene, and each is 90% identical to MemPhiS and 315.3, which carry the *mf4* gene instead (Figure 2).

## Acquisition of Novel Virulence Genes by Global M1T1 Strain and Effect on Virulence

Two virulence genes, *speA2* and *sda1*, were introduced into the M1T1 strain by prophages and are likely to have contributed to its increased fitness and virulence (*19*,*21*). SpeA2 is an important and potent streptococcal superantigen. Although GAS has a rich superantigen repertoire, different strains harbor different combinations of superantigen genes—some are phage encoded, while others are integrated into the bacterial chromosome. Both the global M1T1 strain and its ancestral SF370 (*15*) strain have the superantigen-encoding genes *speF*, *speG*, *speJ*, and *smeZ1*. However, these 2 strains differ in that the global M1T1 strain has *speA2,* whereas SF370 has *speC* (Table)*.* Both SpeA and SpeC are prophage-encoded, whereas the other M1T1 superantigens are chromosomal. Additionally, the clonal M1T1 strain lacks *speH* and *speI*, which are encoded on a single phage in M1 SF370 (*15*,*21*).

**Table:** Genomic differences between M1T1 and M1 SF370*

Difference	M1T1	SF370
Prophages or prophage remnants	SPhinX (*speA*2), MemPhiS (*mf3/mf4*), PhiRamid (*sda1*)	370.1 (*mf2, speC*), 370.2 (*speH, speI*), 370.3 (*mf3*), 370.4 (phage remnant)
Superantigen genes	***speA2***, *speG*, *speJ*, *smeZ1,*	***speC***, *speG*, ***speH***, ***speI***, *speJ*, *smeZ1*
Streptodornases	*mf/spd*, *mf3/spd OR ****mf4/spd4***, ***sda1***	*mf/spd*, ***mf2***, *mf3/spd3*
Other	Insertion sequence (IS1548), SNP in the SLO/NADGH region	

*SNP, single nucleotide polymorphism; SLO, streptolysin O; NADGH, nicotinamide glycohydrolase. Toxin gene names in **boldface** designate genes that are unique to either strain.

Of particular relevance to this discussion is that the *speA* gene was seen in M1 isolates obtained in the early 20th century but had almost vanished from M1 isolates obtained between the 1920s and early 1980s. The loss of *speA* was thought to be one of the main reasons for the sharp decline in severe invasive GAS infections during this time (*23*,*24*). Likewise, the reintroduction of the *speA2* allele in the M1T1 clonal strain in the 1980s prompted speculations that SpeA, and in particular its allelic variant SpeA2, was a major factor in the resurgence of severe invasive GAS infections during that time (*25*). However, additional studies showed that, although the reintroduction of *speA* may have been a factor, the acquisition of other virulence genes by the M1T1 clone is more likely to have had a more profound effect on its increased fitness and virulence in vivo (*20*,*26*,*27*). Nonetheless, the fact that SpeA was missing from most GAS isolates for >50 years suggests that the reintroduction of this superantigen may have increased the risk for persons to have invasive infections because they lack antibodies that neutralize its superantigenic activity. Indeed, the lack of superantigen-neutralizing antibodies has been shown to increase the risk for invasive disease (*28*,*29*).

Sda1, which was also acquired by the M1T1 global strain, is a potent streptodornase (streptococcal nuclease) (*20*) and is not found in most of the other prevalent strains but has been recently reported in an M12 strain (*16*). Streptodornases are secreted extracellular nucleases classically thought to play an important role in virulence by degrading pus (*30*). Every GAS serotype sequenced so far contains >1 streptodornase paralog. These various streptodornases, which differ in the pH optima for their nuclease activity, are likely functionally nonredundant, possibly having different substrate specificity, and may be differentially active in certain host niches or at different times during the infection. The M1T1 clone has, in addition to *sda1*, the chromosomal streptodornase *spd/mf* (alias *speF*) and another phage-encoded streptodornase, *spd3*/*mf3* (or—less frequently—*spd4/mf4*); however, it lacks *spd2*/*mf2* found in the M1 SF370 (*19*,*20*). Despite the presence of multiple DNases in the bacteria, Sda1 has the highest specific activity among the streptococcal nucleases. We showed that the increased activity of Sda1 has resulted from a frame-shift mutation in its C terminus, and when the additional C-terminal sequence of Sdal was deleted, the enzyme activity dropped significantly (*20*).

Sda1, unlike the other nucleases, appears to play a major role in virulence, and inactivating its gene resulted in a dramatic loss of virulence (*26*,*27*,*31*), whereas introducing it into an avirulent strain led to a virulent phenotype (*26*). Sdal protects bacteria against neutrophils (*31*)—which entrap the bacteria in neutrophil extracellular traps (NETs) (*32*)—by degrading these DNA NETs, thereby freeing the bacteria and promoting their ability to invade host tissues (*26*). Additionally, recent evidence suggests that the Sda1 expression may synergize with host factors, leading to additional selective pressure on the bacteria in vivo and resulting in the emergence of a hypervirulent phenotype of the same bacteria (*27*).

Besides the exchange of phage-encoded toxins, additional recombination events may have contributed to the diversification of the M1T1 clone. In a recent study, Sumby et al. (*21*) used DNA–DNA hybridization and single nucleotide polymorphism analysis to show that a 36-kb chromosomal region has been horizontally transferred to M1T1 by recombinatorial replacement from an M12 ancestral strain. This chromosomal region harbors genes encoding 2 important toxins, streptolysin O (SLO) and nicotinamide glycohydrolase (NADGH or NADase), both of which were more highly expressed in M1T1-MGAS5005 compared to M1 SF370 (*21*). Although these differences in expression might be a consequence of the recombination event, we believe that the enhanced expression of these genes is more likely due to a mutation in the *covS* gene of the studied MGAS5005 strain, which resulted in higher expression of virulence networks (*33*). SLO is an important GAS cytolysin that enhances cytotoxicity and toxin translocation (*34*,*35*), and its heightened expression would be expected to increase virulence. It is therefore apparent that several mechanisms led to GAS diversification and that the globally disseminated M1T1 clone has acquired several virulence factors that seem to have contributed to its unusual persistence, spread, and virulence.

## In vivo Selection of Hypervirulent Descendents of Global M1T1 Strain

In addition to the introduction and loss of specific genes in the global M1T1 strain, a high degree of variability in the expression of virulence genes among isolates belonging to this clonal strain was reported (*3*,*36*). This variable expression, in part, depended on where and when the isolates were recovered from the host. However, one of the most notable changes in gene expression that arises in response to host environmental pressure is the remarkable downregulation of the major streptococcal protease, SpeB, and the consequent significant increase in bacterial invasion and severity of GAS sepsis (*27*,*37*,*38*).

Earlier studies by Kansal et al. (*37*) provided the first hint for the reciprocal relation between SpeB expression and severity of GAS sepsis, when they observed that isolates recovered from patients with more severe cases expressed no, or significantly less, SpeB compared to those recovered from patients with nonsevere cases. In ensuing studies, we found that M1T1 regulates its secreted proteins by at least 2 mechanisms (*39*), a transcriptional regulation, and a posttranslational degradation and remodeling of bacterial proteins by SpeB, that, itself, is tightly regulated ( *40*; Technical Appendix, supplementary reference 41). The secreted proteome in the presence and absence of active SpeB is starkly different. Essentially most extracellular virulence factors, including M protein, streptokinase, SpeF, Sda1, C5a peptidase, and the secreted inhibitor of complement, are degraded by this protease, resulting in decreased virulence. The advantage of this massive degradation of virulence factors to the bacteria is not entirely known, but we predict that this may be a means by which the bacteria camouflage themselves from the host during the initial stages of infection. By degrading their virulence components, bacteria may evade initial innate host defenses at the site of the infection until they gain access to a host niche (e.g., skin), where they can start to multiply. Thus, SpeB may facilitate the initial invasion of bacteria through its proteolytic action on host matrix proteins. However, within 60–80 hours after infection, the bacteria are subjected to a hostile human environment, and consequently, there is a selection for more fit mutants within the bacterial community that are better adapted to confront host defenses and gain access to blood and possibly other sterile sites. The more fit mutants, it turns out, are those that lack SpeB expression because of a mutation in *covS*, which is a part of a 2-component regulatory system (CovRS) involved in regulating 15% of GAS genes including SpeB (Technical Appendix, supplementary reference 42). Indeed, recent studies provided evidence for the co-existence of at least 2 very different phenotypic forms of M1T1 bacteria in the initial stages of infection through the skin of mice characterized by SpeB^+^ or SpeB^–^ phenotypes (*27*,*39*).

The downregulation of SpeB spares several key virulence factors that include Sda1 and streptokinase. Our recent studies showed that sparing Sda1 frees the bacteria from neutrophil NETs (*27*). Similarly, in a human plasminogen-transgenic mouse model, sparing streptokinase allowed accumulation of surface plasmin activity and increased bacterial evasion (Technical Appendix, supplementary reference 43). Additional differentially expressed genes in the in vivo–selected *covS* mutants are also likely to contribute to increased virulence, and these are currently being investigated.

## M1T1 and the Future of GAS Epidemiology

Is there an exit plan for M1T1? How long will this strain survive and prevail? Will there be another prevalent strain in the future? It is intriguing that although M1T1 causes deadly conditions, this clone keeps infecting many persons, retaining its superior prevalence. This suggests that there is an exit plan for this clone, or that it is so widely spread among the human population that it keeps being transmitted through genetically protected persons, who serve as reservoirs for it. The diversity within the bacterial population in the host also suggests that while hypervirulent mutants cause deadly diseases when the bacteria invade unusual niches, the less virulent members of the same population survive well in the primary niche (e.g., the throat or nasopharynx) and thus could drive the disease transmission.

Several potentially interactive factors may have contributed to the persistence of M1T1 and may maintain this strain for a long time. These factors include the acquisition of new virulence genes and the differential regulation and expression of virulence genes caused by selection of mutants within the microbial community. These changes in the pathogen, as well as changes in herd immunity and differential host susceptibility, are likely to create dynamic interactions between streptococci and their human host.

When novel strains or clones emerge that express novel proteins or variants of old proteins, these strains are endowed with the ability to better withstand the pressure of herd immunity. According to this hypothesis, M1T1 and other strains that reemerged in the mid-1980s may have successfully survived herd immunity either because they acquired new protein-encoding genes or because they possessed allelic variants of key genes encoding proteins and/or novel alleles that were as-yet unsampled by the immune system. Also, the acquisition of new genes or the sparing of existing proteins from proteolytic degradation may have endowed the bacteria with means to better evade host immune defenses.

In summary, we believe that the emergence of the M1T1 strain, its diversification by phage acquisition, and the in vivo selection of more fit members of its community present an intriguing example of molecular events that can drastically change the epidemiology and virulence of an otherwise avirulent or less virulent organism. Predicting whether other GAS strains may follow a similar trajectory to M1T1 is difficult: The next prevalent strain to emerge may have to combine changes in chromosomal and phage-encoded genes to enhance its fitness and allow it to adapt to different host environments; it also has to be resistant enough to phage-driven lysis. (More prophages enrich the bacteria with additional toxins, but they may also bring the potential risk of lysing the bacteria at any time a phage is induced.) As there are now more and more examples of phage exchange even within and between different bacterial species (Technical Appendix, supplementary reference 44), the traditional classification schema may have to be replaced by ones that better reflect the bacterial virulome. This virulome, as discussed here, can be grossly altered, depending on the environment the bacteria face and the consequent selection of underrepresented minority of the bacterial community that is best adapted to deal with various hostile host milieus.

## Supplementary Material

Technical Appendix Rise and Persistence of Global M1T1 Clone of Streptococcus pyogenes
