# Supplementary material for: Rise and Persistence of Global M1T1 Clone of Streptococcus pyogenes
Source: Emerg Infect Dis. 2008 Oct;14(10):1511–7. doi: 10.3201/eid1410.071660 (PMC2609857; doi:10.3201/eid1410.071660)
Supplement: Technical Appendix — Rise and Persistence of Global M1T1 Clone of Streptococcus pyogenes [file 07-1660_Techapp-s1.pdf]

Rise and Persistence of Global M1T1 Clone of *Streptococcus pyogenes*

Technical Appendix

Table 1. Range of GAS infections:*

A) According to the pathological pattern and mechanism of pathogenesis

Localized	Invasive	Nonsuppurative postinfection sequelae
Pharyngitis	Cellulitis	Acute rheumatic fever
Tonsillitis	Bacteremia	Rheumatic carditis
Impetigo	Meningitis	Acute glomerulonephritis
Otitis media	Myositis	Rheumatic heart disease
Sinusitis	Peritonitis	Sydenham's chorea
Septic arthritis	Scarlet fever	PANDAS
Vaginitis	STSS	
	NF	
Erysipelas		
Other infections†		

B) According to the extent of infection

Asymptomatic	Noninvasive	Invasive	
		Nonsevere	Severe
Colonization	Mucous membranes:	Cellulitis	STSS
	Pharyngitis	Mild bacteremia	NF
	Tonsillitis	Erysipelas	STSS + NF
	Otitis Media	Myositis	Meningitis
	Sinusitis		
	Vaginitis		
	Skin:		
	Impetigo/pyoderma		

*GAS, group A streptococcal; PANDAS, pediatric autoimmune neuropsychiatric disorders associated with *Streptococcus*; STSS, streptococcal toxic-shock syndrome; NF, necrotizing fasciitis.
†GAS can also cause pneumonia, puerperal sepsis, and osteomyelitis.

Table 2. Examples of epidemiological studies performed in the last two decades to characterize GAS diseases*

Where	Period	No. patients	M1T1 percentage	Notes	Ref.
Rocky Mountain Area, USA	1986–1988	20			(*1*)
USA (CDC)	1972–1988	5193	7.1% of all cases and 36% of sepsis cases		(*2*)
USA	1986–1990	34	74% M1 and M3	All were STSS cases	(*3*)
USA	1986–1990	108	28.7%		(*4*)
Pima County, AZ, USA	1985–1990	128	19%	High incidence in Native Americans	(*5*)
New Zealand	1980–1991	9389	7% (became 15.2% in 1984–1985)		(*6*)
Ontario	1991–1993	323† (258 isolates)	24%		(*7*)
Ontario	1991–1995	77†	35%	NF cases	(*8*)
Finland	1988–1995	4597	100%		(*9*)
Sweden	1983–1995	132	20%	92% of M1 were clonal	(*10*)
Japan	1981–1997	128	NA		(*11*)
Israel	1997–1998	409†	1.2% M1	Jewish Orthodox susceptible	(*12*)
Japan	1992–1995	44	33%	M3 isolates: 33%	(*13*)
	1992–1996	55	60%	M3 isolates: 4%	(*13*)

*Most early studies were retrospective, which made them less conclusive. However, some studies (marked by a dagger, †) have been performed prospectively with population–based surveillance in which almost every GAS case was reported, regardless of the case severity. GAS, group A streptococcal; M1, M1 serotype; Ref., reference; CDC, Centers for Disease Control and Prevention; STSS, STSS, streptococcal toxic-shock syndrome; M3, M3 serotype; NF, necrotizing fasciitis; NA, data not available.

Table 3. Studies on M1T1 clonality

No. patients	Where	Period	Typing methods	Name given to clonal M1T1	M1T1 percentage*	Ref.
19 serious, 37 mild	WHO (USA patients)	1979–1990	REA	M1-inv	Serious, 90%; mild, 44%	(*14*)
665	New Zealand	1980–1991	RFLP coupled with PFGE	Type 1a	74%	(*6*)
126	13 countries	NA	RFLP, *emm* typing, MLEE	ET1/RFLP pattern 1a	91% ET1 and 66% ET1s/RFLP 1a	(*15*)
98	Finland	1988–1995	REA, ribotyping, and RAPD,	NA	100% clonal (including 66% identical)	(*9*)
	NC, USA	1994–1995	PFGE	PFGE pattern A	90% during outbreak	(*16*)
39	Japan	1981–1988	PFGE	PFGE2aB1/Biotype 1	0% (0/39)	(*11*)
34		1989–1997			70% (24/34)	

*M1T1, global M1T1 clone; REA, restriction endonuclease; RFLP, restriction fragment length polymorphism; PFGE, pulsed-field gel eletrophoresis; MLEE, multilocus enzyme electrophoresis; NA, data not available.

References

1. Stevens DL, Tanner MH, Winship J, Swarts R, Ries KM, Schlievert PM, et al. Severe group A streptococcal infections associated with a toxic shock–like syndrome and scarlet fever toxin A. N Engl J Med. 1989;321:1–7. PubMed

2. Schwartz B, Facklam RR, Breiman RF. Changing epidemiology of group A streptococcal infection in the USA. Lancet. 1990;336:167–71.

3. Hauser AR, Stevens DL, Kaplan EL, Schlievert PM. Molecular analysis of pyrogenic exotoxins from *Streptococcus pyogenes* isolates associated with toxic shock–like syndrome. J Clin Microbiol. 1991;29:1562–7. PubMed

4. Musser JM, Hauser AR, Kim MH, Schlievert PM, Nelson K, Selander RK. *Streptococcus pyogenes* causing toxic–shock–like syndrome and other invasive diseases: clonal diversity and pyrogenic exotoxin expression. Proc Natl Acad Sci U S A. 1991;88:2668–72. PubMed DOI: 10.1073/pnas.88.7.2668

5. Hoge CW, Schwartz B, Talkington DF, Breiman RF, MacNeill EM, Englender SJ. The changing epidemiology of invasive group A streptococcal infections and the emergence of streptococcal toxic shock–like syndrome. A retrospective population–based study [published erratum appears in JAMA 1993 Apr 7;269(13):1638] [see comments]. JAMA. 1993;269:384–9. PubMed DOI: 10.1001/jama.269.3.384

6. Martin DR, Single LA. Molecular epidemiology of group A streptococcus M type 1 infections. J Infect Dis. 1993;167:1112–7. PubMed

7. Davies HD, McGeer A, Schwartz B, Green K, Cann D, Simor AE, et al. Invasive group A streptococcal infections in Ontario, Canada. N Engl J Med. 1996;335:547–54. PubMed DOI: 10.1056/NEJM199608223350803

8. Kaul R, McGeer A, Low DE, Green K, Schwartz B. Population–based surveillance for group A streptococcal necrotizing fasciitis: Clinical features, prognostic indicators, and microbiologic analysis of seventy–seven cases. Ontario Group A Streptococcal Study. Am J Med. 1997;103:18–24. PubMed DOI: 10.1016/S0002-9343(97)00160-5

9. Muotiala A, Seppala H, Huovinen P, Vuopio–Varkila J. Molecular comparison of group A streptococci of T1M1 serotype from invasive and noninvasive infections in Finland. J Infect Dis. 1997;175:392–9. PubMed

10. Eriksson BK, Andersson J, Holm SE, Norgren M. Invasive group A streptococcal infections: T1M1 isolates expressing pyrogenic exotoxins A and B in combination with selective lack of toxin–neutralizing antibodies are associated with increased risk of streptococcal toxic shock syndrome. J Infect Dis. 1999;180:410–8. PubMed DOI: 10.1086/314872

11. Murase T, Suzuki R, Osawa R, Yamai S. Characteristics of Streptococcus pyogenes serotype M1 and M3 isolates from patients in Japan from 1981 to 1997. J Clin Microbiol. 1999;37:4131–4. PubMed

12. Moses AE, Goldberg S, Korenman Z, Ravins M, Hanski E, Shapiro M. Invasive group A streptococcal infections, Israel. Emerg Infect Dis. 2002;8:421–6. PubMed

13. Ikebe T, Murai N, Endo M, Okuno R, Murayama S, Saitoh K, et al. Changing prevalent T serotypes and *emm* genotypes of *Streptococcus pyogenes* isolates from streptococcal toxic shock–like syndrome (TSLS) patients in Japan. Epidemiol Infect. 2003;130:569–72. PubMed

14. Cleary PP, Kaplan EL, Handley JP, Wlazlo A, Kim MH, Hauser AR, et al. Clonal basis for resurgence of serious *Streptococcus pyogenes* disease in the 1980s. Lancet. 1992;339:518–21. PubMed DOI: 10.1016/0140-6736(92)90339-5

15. Musser JM, Kapur V, Szeto J, Pan X, Swanson DS, Martin DR. Genetic diversity and relationships among Streptococcus pyogenes strains expressing serotype M1 protein: recent intercontinental spread of a subclone causing episodes of invasive disease. Infect Immun. 1995;63:994–1003. PubMed

16. Kiska DL, Thiede B, Caracciolo J, Jordan M, Johnson D, Kaplan EL, et al. Invasive group A streptococcal infections in North Carolina: epidemiology, clinical features, and genetic and serotype analysis of causative organisms. J Infect Dis. 1997;176:992–1000. PubMed

Supplementary References

41. Kreikemeyer B, McIver KS, Podbielski A. Virulence factor regulation and regulatory networks in *Streptococcus pyogenes* and their impact on pathogen–host interactions. Trends Microbiol. 2003;11:224–32. PubMed

42. Graham MR, Smoot LM, Migliaccio CA, Virtaneva K, Sturdevant DE, Porcella SF, et al. Virulence control in group A *Streptococcus* by a two–component gene regulatory system: global expression profiling and in vivo infection modeling. Proc Natl Acad Sci U S A. 2002;99:13855–60. PubMed DOI: 10.1073/pnas.202353699

43. Walker MJ, McArthur JD, McKay F, Ranson M. Is plasminogen deployed as a *Streptococcus pyogenes* virulence factor? Trends Microbiol. 2005;13:308–13. PubMed DOI: 10.1016/j.tim.2005.05.006

44. Davies MR, Tran TN, McMillan DJ, Gardiner DL, Currie BJ, Sriprakash KS. Inter–species genetic movement may blur the epidemiology of streptococcal diseases in endemic regions. Microbes Infect. 2005;7:1128–38. PubMed DOI: 10.1016/j.micinf.2005.03.018
